# Formation Kinetics and Antimicrobial Activity of Silver Nanoparticle Dispersions Based on N-Reacetylated Oligochitosan Solutions for Biomedical Applications

**DOI:** 10.3390/pharmaceutics15122690

**Published:** 2023-11-28

**Authors:** Ekaterina K. Urodkova, Ol’ga Ya. Uryupina, Vladimir E. Tikhonov, Natalia E. Grammatikova, Anastasia V. Bol’shakova, Anna A. Sinelshchikova, Alexandra I. Zvyagina, Dmitry N. Khmelenin, Elena S. Zhavoronok, Ivan N. Senchikhin

**Affiliations:** 1 A.N. Frumkin Institute of Physical Chemistry and Electrochemistry, Russian Academy of Sciences, 119071 Moscow, Russia; urupina635@mail.ru (O.Y.U.);; 2 A.N. Nesmeyanov Institute of Organoelement Compounds, Russian Academy of Sciences, 119991 Moscow, Russia; tikhon@ineos.ac.ru; 3 G.F. Gause Institute of New Antibiotics, 119021 Moscow, Russia; ngrammatikova@yandex.ru; 4 A.V. Shubnikov Institute of Crystallography, Russian Academy of Sciences, 119333 Moscow, Russia; 5 Lomonosov Institute of Fine Chemical Technologies, MIREA—Russian Technological University, 119571 Moscow, Russia

**Keywords:** chitosan, oligochitosan, hydrochloride, silver nanoparticles, surface functionalization, antimicrobial activity

## Abstract

The paper presents the results of the synthesis, a detailed kinetics study, and an investigation of the biological activity of silver nanoparticles (AgNPs) in aqueous solutions of N-reacetylated oligochitosan hydrochloride. UV–visible spectrophotometry and dynamic light scattering were employed to control silver ion reduction. The process was observed to follow a pseudo-first-order law. Transmission and scanning electron microscopy demonstrated that AgNPs ranging in size from 10 to 25 nm formed aggregates measuring 60 to 90 nm, with the aggregate surface coated by a 2–4 nm chitosan shell. X-ray microanalysis and powder X-ray diffractometry were used to study the phase composition, identifying two crystalline phases, nanocrystalline silver and AgCl, present in the dispersions. The antibacterial effect was assessed using the serial dilution method for dispersions with varying degrees of Ag^+^ conversion. Nanodispersions exhibited significant activity against *Escherichia coli*, *Pseudomonas aeruginosa*, *Bacillus cereus*, and *Staphylococcus aureus*. Interestingly, the activity did not appear to be heavily influenced by the presence of the AgCl phase or the concentration of Ag^+^ ions. These synthesized dispersions hold promise for the development of materials tailored for biomedical applications.

## 1. Introduction 

One of the foremost challenges in modern healthcare is antibiotic resistance [1,2,3,4,5,6]. The discovery of antibacterial medicines represented a significant breakthrough, enabling the extension of the lifespans of multiple generations [7,8,9,10]. However, overuse of pharmaceuticals has contributed to the emergence of resistance among pathogens. The development of novel antibiotics is currently constrained, necessitating an active quest for alternative remedies that could be an alternative to antibiotics. Nanostructures based on noble metals and their compounds stand out as promising candidates for such therapeutics [11,12,13,14,15,16,17]. These nanoparticles (NPs), with their ability to interact with cells [12,18,19], can infiltrate them and disrupt the acid–alkaline balance of bacteria, thereby disturbing their homeostasis [12].

Silver nanoparticles (AgNPs) are particularly notable for their potent antibacterial properties [12,20,21,22,23,24], and they are already widely employed as antimicrobial agents [25]. However, it is worth noting that AgNPs, when present in concentrations above a certain threshold, can exhibit cytotoxicity towards human cells [26,27]. This cytotoxicity may lead to DNA damage, chromosomal aberrations, and cell death. Consequently, there remains a pressing need to engineer AgNP forms [18] that exhibit reduced cytotoxicity while retaining high antimicrobial efficacy. To achieve this, AgNPs are synthesized with a protective polymer layer that not only controls the size and shape but also stabilizes their dispersions [27]. Furthermore, the polymer can act as an NP stabilizer but also as a reducer of silver ions. AgNPs synthesized in chitosan solutions can be an example of such systems (see reviews [28,29,30] and references therein).

Chitosan and its derivatives have sustained considerable interest for several decades due to its diverse properties that hold significance for medical applications [31,32,33,34]. Chitosan is actively utilized in the development of medical products, including wound dressings [35,36] and burn wound treatments [37,38], dental compositions [39], drug delivery systems [40,41], dietary supplements [42], and various other applications. At the same time, chitosan-based systems have been actively used as a medium for the synthesis of silver nanoparticles [43,44,45,46,47,48,49,50,51,52,53], including aqueous solutions of chitosan [46,47,49,50,51,52,54].

It is noted that the higher the MM of chitosan, the finer the size of silver nanoparticles obtained (up to 8 [46] or even 2–3 nm [55], which significantly increases the probability of their cytotoxic properties [46]). In addition, it should be realized that chitosan is a natural biopolymer with a rather broad molecular weight distribution, which is still probably broadened in the processes of its production from chitin, which are statistical in nature [56,57]. Finally, high molecular weight chitosans derived from chitin under conditions of heterogeneous deacetylation are characterized by a nonregular distribution of residual acetyl groups within the macromolecules, which does not always ensure reproducible properties [58,59]. This is all the more important because, according to [28], the reducing and stabilizing properties of chitosan seem to be related to the presence of NH_2_ groups in the polymer chain, including those resulting from deacetylation.

From this point of view, oligomeric forms of chitosan—oligochitosans (OChTs) with molecular masses from 2 to 16 kDa are of great interest [60,61,62,63,64,65]. The lower MM and narrower molecular weight distribution of OChTs provide several advantages related to their improved solubility in water, the relatively low viscosity of the resulting aqueous solutions, and better compatibility with many components of pharmaceutical and other compositions [58,66,67]. In addition, there are reports that oligochitosans have enhanced antimicrobial activity [58,61,65], and the transition from high molecular weight chitosan to oligomeric chitosan markedly improves its reducing ability to silver ions [50,51]. It is of interest to note that, according to [50], oligomeric chitosans ensure the stability of silver nanoparticles due to steric and electrostatic factors, while chitosans with medium MM, on the contrary, act as flocculants, which leads to aggregation and sedimentation of particles during storage. At the same time, the problem associated with the heterogeneous distribution of acetyl groups along the macromolecular chain can be solved by homogeneous reacetylation, initially proposed in [68]. Through this method, it becomes feasible to produce water-soluble reacetylated forms of OChTs while maintaining a specific range of molecular weights, even up to pH values of 12.5 [58]. Nevertheless, the works devoted to the synthesis of silver nanoparticles in the medium of oligochitosans with a regular distribution of N-acetyl groups along the macromolecular chain are practically absent. Thus, the synthesis of silver nanoparticles of adjustable size in aqueous solutions of reacetylated OChTs is of specific scientific interest and also has good opportunities for applications.

For commercial production of biomedical products based on nanodispersions of silver particles, fine control of synthesis conditions and quality of the resulting product is very important. The conditions of NP synthesis, the composition of systems, purity, and non-toxicity of the final product are of great significance in the production of medical supplies. Most often, the synthesis of silver nanodispersions in chitosan and its derivatives’ mediums involves the addition of other components, which can potentially contaminate the surface of NPs, necessitating the need for cleaning procedures (see [27] and the cited literature). In a prior study, we proposed [69] a simple and reproducible method for the synthesis of AgNPs using oligochitosan as a reducing agent and stabilizer. In [69], we successfully obtained dispersions of relatively large particles (40–70 nm) in aqueous OChT solutions without the use of additional reducers and stabilizers. In essence, OChT served as both the reducer of silver ions and the stabilizer for the formed AgNPs. The resulting AgNPs contain amine groups on the surface, which greatly facilitates their further functionalization. The possibility of achieving stable dispersions with a narrow size distribution was demonstrated for oligomers of varying molecular weights and degrees of acetylation [69]. Among the OChTs examined was N-reacetylated OChT. In a subsequent study [70], it was reported that the narrowest AgNP size distribution was achieved in N-reacetylated chitosan dispersions. Furthermore, these dispersions exhibited exceptional stability over time.

Building upon these findings, this work aims to investigate in great depth the formation of stable dispersions of sufficiently large AgNPs in N-reacetylated oligochitosan (OChT-R) medium to obtain kinetically and aggregatively stable systems that can be used for biomedical applications. Specifically, the study will explore the kinetics of AgNP formation in aqueous OChT-R solutions, analyze AgNP structure and shape, assess phase composition, and evaluate their antimicrobial activity and the possibility of practical application for certain fields.

## 2. Experimental Section

### 2.1. Materials

N-reacetylated chitosan hydrochloride with a molecular weight (MW) of 12 kDa and degree of acetylation (DA) of 24 mol.% (OChT-12/24-R) was selected for this study. OChT was prepared according to the procedure outlined in [58] using the high-molecular-weight HMW (MW = 350 kDa, DA = 5%) chitosan produced by LLC Bioprogress, Russia in two stages: (1) partial depolymerization of HMW chitosan and preparation of oligochitosan (OChT), and (2) partial N-reacetylation of oligochitosan.

Depolymerization of HMW chitosan was carried out by the acid hydrolysis of HMW chitosan in 0.6 M hydrochloric acid solution at 70 °C. OChT hydrochloride was precipitated using ethanol and dried under vacuum over sodium hydroxide till a constant weight was achieved as described in [66]. N-reacetylation of OChT was carried out following the protocol published in [58]. Shortly, the OChT sample was dissolved in the water, and the pH of the solution was adjusted to 6.0 with a 0.1 M sodium hydroxide solution under intensive stirring. Then, the solution was diluted with an equal volume of methanol and cooled to 20 °C. Acetic anhydride was added in the required amount, and a concentrated ammonia solution was added after one hour. The mixture was dialyzed against deionized Milli-Q water using a dialysis tube with a cutoff of 1 kDa for several days. Half of the dialyzed solution was lyophilized, and the basic form of N-reacetylated oligochitosan (OChT-R*) was collected. Afterward, hydrochloric acid was added to the second part of the dialyzed solution up to pH 3.0. This solution was lyophilized, and the hydrochloric form of N-reacetylated oligochitosan (OChT-R) was collected. As a result, the basic and hydrochloric forms of N-reacetylated oligochitosan were prepared. Molecular characteristics (MW ± 0.5 kDA and DA ± 1%) were determined by HP-SEC and ^1^HNMR methods [58]. As found, both the basic form of reacetylated oligochitosan (OChT-12/25-R*) and its hydrochloric form (OChT-12/24-R) had the same molecular weight and practically similar degrees of N-acetylation (25 and 24%, correspondingly). Both forms were used in this study.

Silver nitrate (99.9%, Aldrich) was applied as a precursor. The pH of the reaction mixture for NP synthesis was controlled by adding an aqueous solution of Na_2_CO_3_ (analytical grade). All solutions were prepared on the day of the synthesis in freshly double-distilled water deionized on Milli-Q Synthesis (Millipore Corp., Burlington, MA, USA) at a resistivity of 18.4 mΩ/cm and temperature of 23 °C. All glassware used in the synthesis was prewashed with a mixture of concentrated hydrochloric and nitric acids and repeatedly rinsed with distilled water.

### 2.2. Methods

The synthesis of NPs in the medium of N-reacetylated chitosan solution followed the procedure described in [69], with the main steps outlined as follows: a 0.05 wt.% aqueous solution of OChT was introduced into a predetermined volume of deionized double-distilled water, and the mixture was heated to 75 °C under intensive stirring using a magnetic stirrer. Then, a 0.17 wt.% aqueous solution of AgNO_3_ and a 0.5 wt.% aqueous solution of Na_2_CO_3_ were added consistently at specific time intervals under constant stirring.

The control of NP formation and determination of their average size were conducted using dynamic light scattering (DLS, a laser wavelength of 633 nm) using Zetasizer Nano ZS (Malvern, UK) and absorption spectroscopy in the ultraviolet and visible regions with Evolution 300 (Thermo Electron Corp., Waltham, MA, USA).

The pH of the reaction systems was measured using an MPT-2 autotitrator (the optional module of the Zetasizer Nano ZS).

Powder X-ray diffractometry (PXRD) was applied to evaluate the composition and microstructural characteristics of the synthesized NPs. The measurements were performed using an Empyrean (PANalytical B.V., Almelo, The Netherlands) diffractometer: CuKα1 = 1.5405 Å, diffracted beam, time per step 300 s, step size 0.033°, Bragg–Brentano geometry.

Structural and morphological characteristics of AgNPs were investigated by scanning and transmission electron microscopy. 

The sizes and morphologies of NPs were determined by scanning electron microscopy (SEM) using a Quanta 650 FEG microscope (Thermo Fisher Scientific, Waltham, MA, USA) equipped with an Octane Elect Plus energy dispersive X-ray (EDX) detector (EDAX, Pleasanton, CA, USA). Subsequently, preliminarily dispersions were washed repeatedly by centrifugation. After that, a droplet of dispersion was placed onto a silicon support, allowed to evaporate completely, and then placed into an instrument chamber for examination under a high vacuum using an accelerating voltage of 5–20 kV. X-ray spectral analysis was performed at an accelerating voltage of 30 kV. The spectra were analyzed by applying the original EDAX Genesis software V6.54.

The size and structure of the synthesized NPs were analyzed by transmission electron microscopy (TEM) using an LEO 912 AB Omega microscope (Carl Zeiss, Oberkochen, Germany) and an Osiris high-resolution transmission electron microscope (HRTEM, Thermo Fisher Scientific, Waltham, MA, USA). For this purpose, drops of aqueous dispersions of AgNPs were dropped onto copper grids and dried for several minutes.

The antibacterial activity of silver nanodispersions based on solutions of N-reacetylated OChT was studied against *Escherichia coli* ATCC 25922, *Pseudomonas aeruginosa* ATCC 27853, *Bacillus cereus* ATCC 10702, *Staphylococcus aureus* ATCC 29213. The dispersions with maximum silver concentrations (about 300 μg/mL) were chosen to evaluate the antibacterial activity of the dispersion.

Minimal inhibitory concentrations (MICs) were determined by serial microdilutions in Mueller–Hinton broth according to the guidelines [71].

For this purpose, the initial dispersions were diluted to the required concentration using Mueller–Hinton broth (256 µg/mL). The inoculum was prepared from a standardized microbial suspension adjusted to 0.5 on the McFarland scale and further diluted to 10^5^ CFU/mL when performing the test. The experiment was conducted with 96-well sterile plastic plates, and MICs were determined after 18–22 h of growth at 36 ± 1 °C. MICs are the concentrations at which no visible growth of microorganisms is present in the well of the plate.

## 3. Results and Discussion

### 3.1. Synthesis and Characteristics

Dispersions of AgNPs were obtained according to the protocol [69] in the medium of an aqueous solution of N-reacetylated OChT. It was shown [69,70] by IR spectroscopy that chitosan acted in this process as a silver ion reducer and AgNP stabilizer due to hydroxyl and amino groups.

According to [69], the monomodal particle distribution was achieved over a wide range of concentration ratios of initial reagents in the case of N-reacetylated OChT against dispersions based on other studied OChTs. Moreover, dispersions had better stability over time (the number, mean size, and relative numerical concentration of particles were stable for about 1.5 months instead of 4 weeks). The average size of AgNPs was 68–77 nm, depending on the synthesis conditions. The reaction time ranged from 50 min for a reaction mixture with pH 6.8 to 15 min for a system with pH 8.3 [69]. During this time, the initially colorless solution turned yellow. Next, the color saturation increased, and the system gradually became reddish-brown. At this time, the reaction was halted by cooling the dispersion in a refrigerator compartment (4 °C). The quantification of precursor reduction was discussed in detail in [69].

In the present work, the kinetics of silver ion reduction in OChT solutions was investigated to estimate their conversion degree α. For this purpose, the synthesis of AgNPs in the medium of N-reacetylated OChT occurred at the following content of components in the reaction system with the volume ratio H_2_O/OChT-12/24-R(0.05%)/AgNO_3_(0.17%)/Na_2_CO_3_(0.5%) = 25.5/10/8/0.5. According to previous experiments, dispersions were formed with monomodal particle size distribution at this component ratio, and they exhibited one of the lowest values of the coefficients of variation *C*_v_ equal to 2.0. Moreover, this system had the maximum silver content (about 300 μg/mL). Finally, the silver reduction rate at these component ratios was visually relatively high.

It is noted in [58,59] that chitosans reacetylated under homogeneous conditions exhibit a statistical distribution of N-acetylglucosamine units in the chain, in contrast to chitosans deacetylated under heterogeneous conditions [72]. Consequently, a more homogeneous distribution of functional groups in the OChT molecule may promote a more uniform formation of NPs and, thus, a narrower distribution of AgNP size.

**Figure 1:**
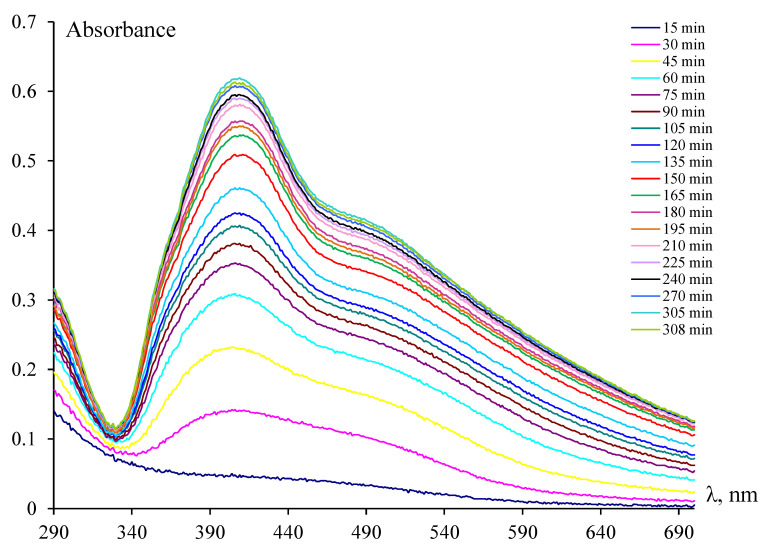
The evolution of the absorption spectra during Ag^+^ reduction.

Figure 1 illustrates the evolution of the absorption spectra during Ag^+^ reduction. Notably, visible changes, characterized by an absorption band with a peak at 405 nm, corresponding to localized surface plasmon resonance (LSPR), and a broad shoulder near 500 nm of slightly lower intensity, began to appear only after a time τ = 30 min after the initiation of the process.

Thus, within the range of τ values from 30 to 60 min, the intensity of the LSPR band exhibited a sharp contrast. The value of the absorption maximum underwent an almost threefold change, rising from 0.13 to 0.35. At the same time, the absorption band maximum shifted slightly to the long-wave region (from 405 to 409 nm). During the following time frame (*τ* from 60 min to 3 h from the beginning of the process), the growth of the absorption intensity of the LSPR band significantly decreased and was virtually completed after 5 h of synthesis. In this scenario, the absorption intensity at the 405 nm maximum of the band and the long-wave shoulder (near 500 nm) were nearly equivalent during the initial period of the reaction. Then, as τ increased, the intensity of the band at 409 nm increased at a faster rate than that of the long-wave shoulder, exceeding it by approximately 1.5 times by the end of the synthesis. The presence of the LPPR band in the UV–visible spectrum unequivocally indicates the formation of AgNPs [73].

In this manner, the reduction of silver ions proceeded over an extended period. Moreover, during the initial 15 min of the process, the surface plasmon resonance peak was not detected. Subsequently, there was a noticeable acceleration of the process, culminating in its eventual termination. In essence, what could be observed here is a typical scenario of particle formation kinetics during chemical reduction in a solution, characterized by an induction period, acceleration, and the subsequent decay of the process [74].

The dependence of the conversion degree of Ag^+^ → Ag^0^ α on the time τ was calculated by the formula:

$$\alpha =\frac{A-A_{0}}{A_{max}-A_{o}},$$

*A_0_*, *A*, *A_max_* are the initial, current, and final values of the absorption peak maximum (Figure 1).

**Figure 2:**
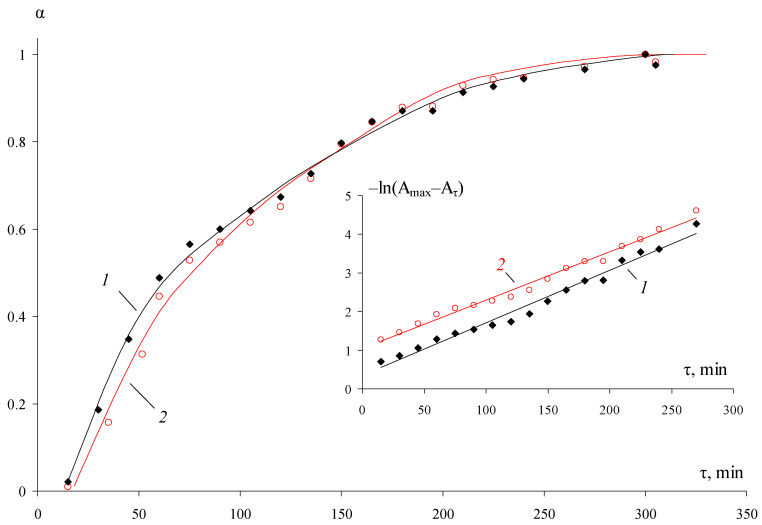
Kinetic dependence of the conversion degree of Ag^+^ → Ag^0^ calculated from the localized SPR peak (1) and the maximum of the long-wave shoulder (2). The inset shows the kinetic dependences −ln(*A_max_* − *A*_τ_).

In addition, Figure 2 shows the dependencies of the conversion degree for the long-wave shoulder of the absorption spectra, as well as the functions −ln(*A*_*max*_ − *A*_τ_) = f(τ) for the maximum at 405–411 nm and the shoulder near 500 nm. It is evident that the dependencies −ln(*A*_*max*_ − *A*_τ_) = f(τ) are linear. It corresponds to the pseudo-first-order reaction. The values of the calculated constants are close to each other and are ≈0.012 min^−1^. The dependence of the maximum of the LSPR peak (*A*_τ_) on the inverse time (Figure S1) allows estimating the extrapolated value $A_{max}^{/}$, corresponding to the end of the Ag^+^ reduction process: $A_{max}^{/}$ ≈ 0.57 and agrees with the experimental value (0.56).

**Figure 3:**
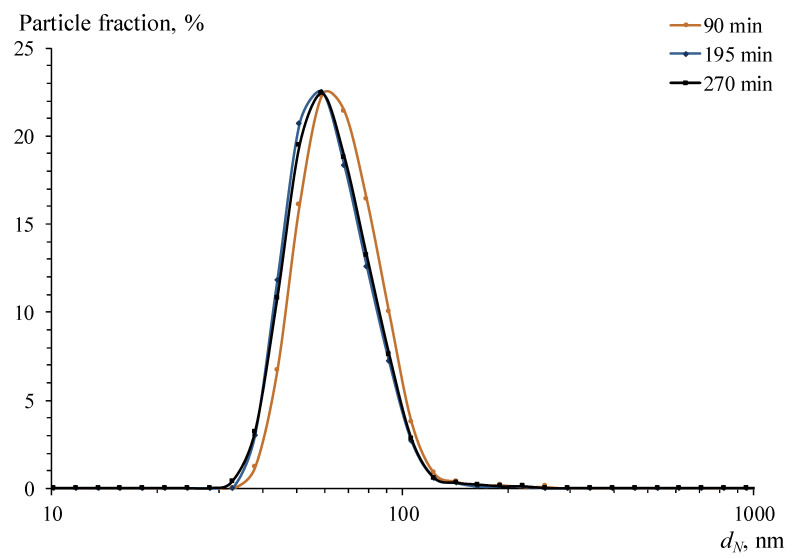
Typical particle size distributions for the system at times 90, 195, and 270 min from the beginning of synthesis.

**Figure 4:**
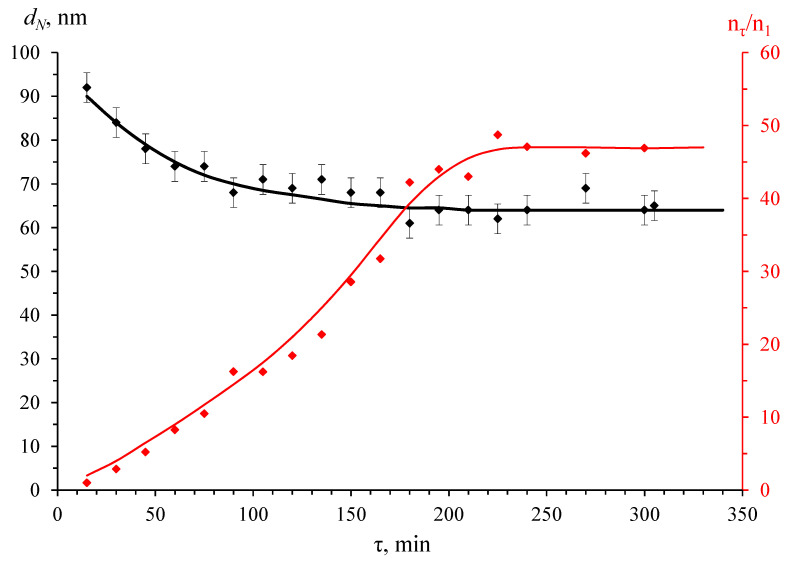
The kinetic dependence of the average numerical size of silver nanoparticles and the relative numerical concentration of nanoparticles on the silver reduction time.

The obtained data illustrate that the time of complete Ag^+^ reduction tends to *τ* = 5 h for this process. The DLS data also showed it (Figure 3 and Figure 4), according to which the average size and relative concentration of particles calculated according to the approach proposed by Vysotskii et al. [75] after 90 min of synthesis remained virtually unchanged. Significantly, the particle size distributions (Figure 3) were strictly monomodal throughout the synthesis.

It is known that the position of the plasmon resonance band maximum of NPs depends greatly on the particle size, polarizability, and dielectric permittivity [27,76,77,78,79,80], so the literature provides different data on the dependence of the size of AgNPs on the maximum of the LSPR peak [81,82,83,84,85]. Additional factors affecting the position of the LSRP peak are the components sorbed on the particle surface, size, and conformational state of the macromolecules stabilizing the NPs. Thus, according to [46], where the dependence of AgNP size on the molecular weight of chitosan and its conformation in synthesis initiated by UV irradiation was investigated, it was found that higher molecular weight chitosan led to the production of smaller AgNPs. For example, for chitosan with a MW of 40 kDa determined by the viscosimetry and calculated from the Mark–Kuhn–Houwink ratio (k = 3.41 × 10^−5^, α = 1.02 [46]), the NP size was 12 nm, and the maximum of the plasmon band in the spectrum was localized at 424 nm. At the same time, for chitosan with an MW of 240 kDa, the particle size was 8 nm, and the plasmon maximum was near 383 nm. Moreover, the authors of [46] demonstrated the dependence maxima of the LSPR on chitosan in dispersion. It turned out that the AgNP LSPR band shifted to a more short-wave region in the case of the tangle conformation compared to the helical one (for chitosan with an MW of 220 kDa, the shift was 7 nm when the pH of the studied dispersion was changed from 4.8 to 3.3). On the other hand, the authors [81] calculated that aqueous dispersions of spherical AgNPs with a size of 40 nm should have an LSPR band with a maximum of 410 nm in the spectrum.

When analyzing the data in Figure 4, the presence of large enough light-scattering centers already at the initial (up to 30 min) stage of synthesis attracts attention. At the same time, there was no LSPR peak in the UV–visible spectra (Figure 1). A possible explanation for this experimental fact will be provided below.

The absorption maximum, observed at 409 nm (Figure 1), probably corresponded to the LSPR of spherical AgNPs with an average diameter of about 15 ± 5 nm, according to [86], and the long-wave shoulder was associated with the absorption of larger particles. It appears that these data challenged the results of average particle size measurements of AgNPs by DLS. As shown in Figure 3 and Figure 4, NPs were formed in the OChT-12/24-R solution with a monomodal and relatively narrow particle size distribution during the entire synthesis. The average size of synthesized AgNPs in the final dispersion was 70 nm.

**Figure 5:**
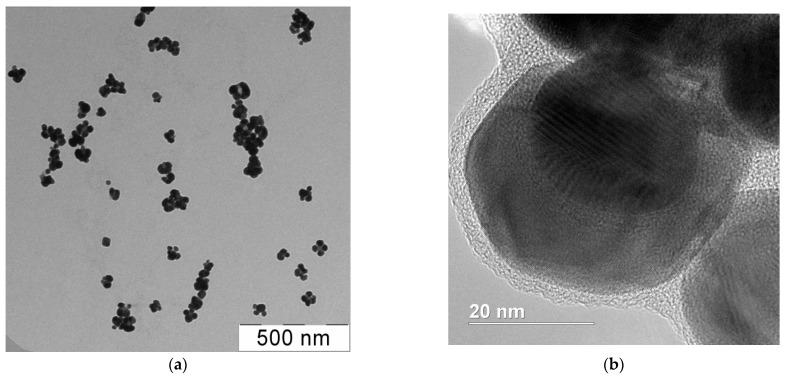
TEM (**a**) and HRTEM-images (**b**) of silver NPs obtained in OChT-12/24-R solution.

The electron microscopy data made it possible to solve the observed mismatch of particle sizes obtained by DLS and UV–visible spectrophotometry methods. The TEM images of the highly reduced (α → 1) dispersions (Figure 5a) show that 10–25 nm single particles formed the aggregates consisting of several individual NPs. The size of these aggregates closely matched that determined by DLS. Remarkably, these data were congruent with the results of SEM (Figure S2). In turn, the application of HRTEM (Figure 5b) made it possible not only to confirm the data obtained by the TEM and SEM methods but also to estimate the thickness (≈2–4 nm) of the polymer shell that covers a particle.

**Figure 6:**
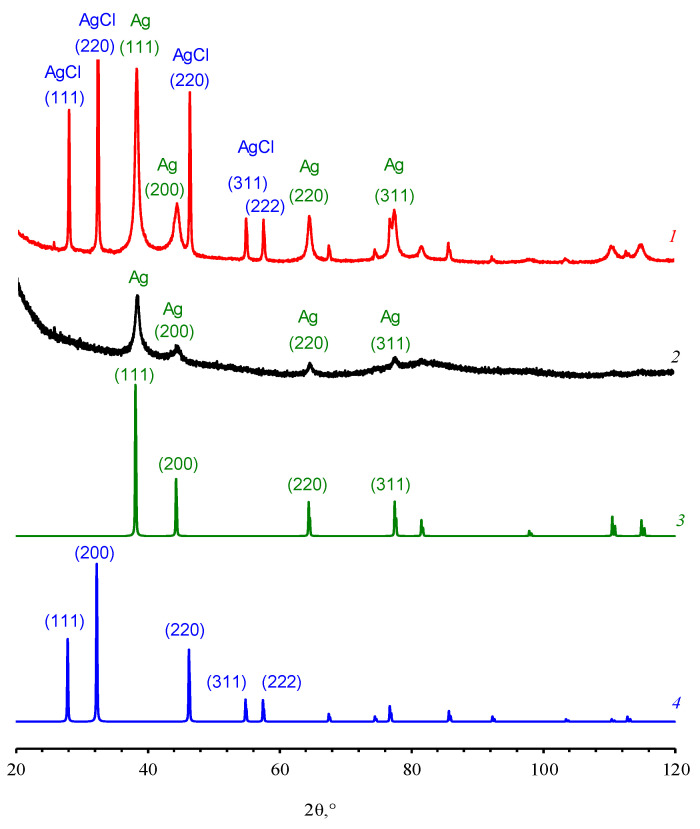
PXRD data for powder samples obtained from dried silver nanodispersions based on OChT-12/24-R solution (1) and OChT-12/25-R* (2) and PDF2 database PXRD patterns of Ag (PDF Number 4-783) (3) and AgCl (PDF Number 31-1238) (4) cubic unit cells in the space group Fm-3m.

Experimental studies of the powders obtained from dried silver nanodispersions by PXRD (Figure 6) showed that the samples contained two crystalline phases. It is important to note that one of the phases in Figure 6 was nanocrystalline (Phase I, Table S1).

Phase I was identified as nanocrystalline silver, crystallized in Fm3m (Z = 4) with a = 4.085 Å and Cu-type structure and *D*_II_ ≈ 40 Å. The analysis of Phase II was of particular interest. It appeared (Figure 6, Table S1) that Phase II was the Fm3m cubic phase (Z = 4) with a = 5.549 Å and NaCl-type structure equated to silver chloride AgCl. The average size of the coherent scattering regions, determined by the XPowderX program using Scherrer’s formula, in Phase II *D*_I_ was ≈360 Å.

Since Phase II was identified as AgCl, the control experiment was performed. A sample of dispersion based on chloride-free OChT (OChT-12/25-R* in Table S1), which was similar to OChT-12/24-R in all its characteristics, was investigated by PXRD. It is important to note that OChT-12/25-R* does not contain chlorine atoms/ions [58] (spectrum 2 in Figure S3). The method [69] was used for AgNP synthesis similar to dispersions based on OChT-12/24-R. As a result, silver dispersions (Figures S4–S6) were obtained and characterized. The thickness of the stabilizing shell on the NPs (Figure S6) was comparable (1–4 nm) to that in the case of particles synthesized in an OChT-12/24-R medium. It turned out (Figure 6) that Phase II was absent on the diffractogram. This fact indirectly confirms Phase II is AgCl. At the same time, the phase of nanocrystalline silver (Phase I) is present. EDX data also argue that silver chloride is formed during the synthesis of AgNPs in OChT-12/24-R solutions.

Chloride is present in the original OChT-12/24-R (spectrum 1 in Figure S3) and in the powder obtained by evaporation of the dispersion (spectrum 3 in Figure S3). However, it is undetectable in the original OChT-12/25-R* (spectrum 1 in Figure S3). According to the authors’ estimates, the concentration of chloride ions in the initial solution of OChT-12/24-R is sufficient for AgCl formation in the reaction volume. Thus, the product of Cl^−^ and Ag^+^ ion concentrations (≈2.5 × 10^−7^) was approximately ten and one hundred times higher than the solubility product *k_sp_* AgCl at 100 °C (3.65 × 10^−8^) [87] and 50 °C (1.40 × 10^−9^) [87], respectively. It could be supposed that when a silver nitrate solution was added to the OChT solution, the silver chloride phase formed at the first stages of AgNP synthesis, and the salt could serve as a nucleating center for the formation of silver particles. However, in the authors’ opinion, the silver chloride phase was likely formed on the surface of AgNPs at the initial synthesis stages. It partly explains the formation of dispersions with large particles at the initial stage of dispersion synthesis (Figure 4), characterized by the absence of a definite LSRP peak (Figure 1). A dense and thick salt shell was likely formed on the metal surface, which led to a corresponding change in the dielectric permittivity of the medium. Further, as chloride anions decreased in number (the concentration was much less than that of Ag^+^), silver particle formation occurred with a less dense polymer stabilizing shell. This led to a shift in the particle distribution towards smaller sizes and the appearance of the LSPR band in the UV–visible spectra. Future reports will delve into a more detailed study of the phase composition and particle structure in the obtained dispersions. For now, let us note one more remarkable fact. The obtained results of qualitative PXRD analysis indicated that during the synthesis of AgNPs according to the proposed method [69], no probable by-product was formed—poorly water-soluble silver carbonate (*k_sp_* = 8.1 × 10^–12^ at 25 °C [88]) (see, for example, experimentally observed Ag_2_CO_3_ diffraction patterns 26-339 (PDF2 database) or mp-4691 and mp-560717 (materialsproject.org).

Thus, at this stage of the work, the formation of AgNPs with an average size of 70 nm in an aqueous solution of N-reacetylated OChT, where OChT acts as both a silver reducer and a stabilizer of NPs, was investigated. The obtained dispersions may be interesting for radiopaque diagnostic systems [89] and antibacterial agent development. Since the obtained nanodispersions contained not only polycrystalline AgNPs but also a silver chloride phase with its biocidal activity [90,91,92], it seems reasonable to investigate and compare the antibacterial activity of dispersions based on OChT-12/24-R and OChT-12/25-R*.

### 3.2. Antibacterial Activity of Dispersions 

**Figure 7:**
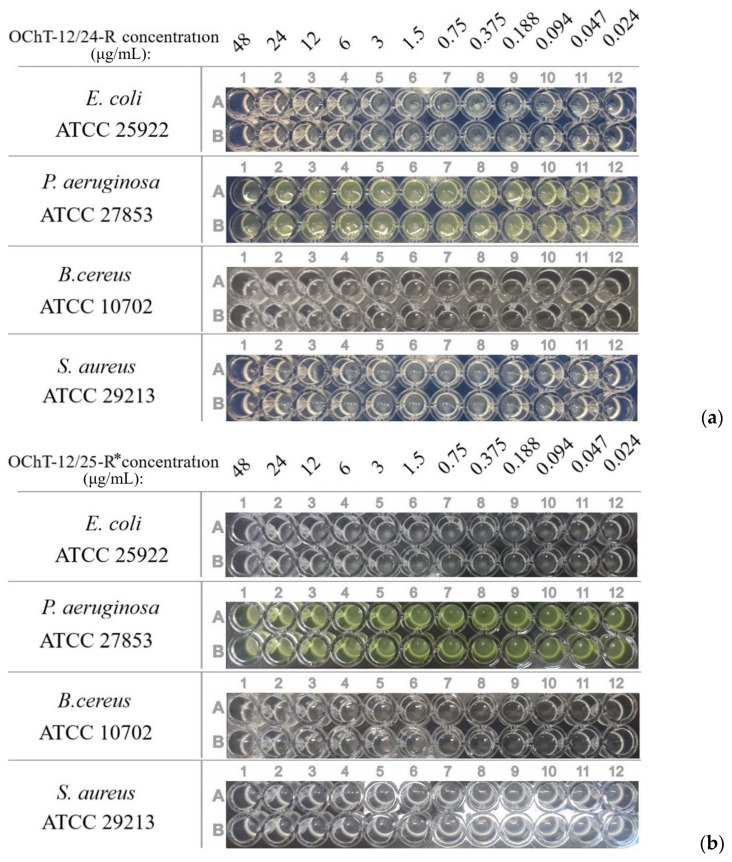
MICs of OChT-12/24-R (**a**) and OChT-12/25-R* (**b**) solutions.

In the first stage, the activity of the initial solutions of OChT-12/24-R and OChT-12/25-R* with the concentration corresponding to the oligomer content in the dispersions were evaluated (Figure 7, Table 1). The experiment showed that the initial solutions with such low concentrations of oligomers did not exhibit significant activity against these strains. Bacterial growth was in all plate wells. Therefore, OChT-12/24-R and OChT-12/25-R* did not contribute significantly to the overall activity of the dispersion.

The systems with a reduction degree α ≈ 45% were chosen to evaluate the antibacterial activity of dispersions based on N-reacetylated hydrochloride OChT-12/24-R and native OChT OChT-12/25-R*. These dispersions, as in the case of non-reacetylated OChTs [70], were the most sedimentation stable and retained their composition and properties for at least 45 days when stored at 4 °C. The ratios of components in dispersions based on different OChTs were similar.

**Figure 8:**
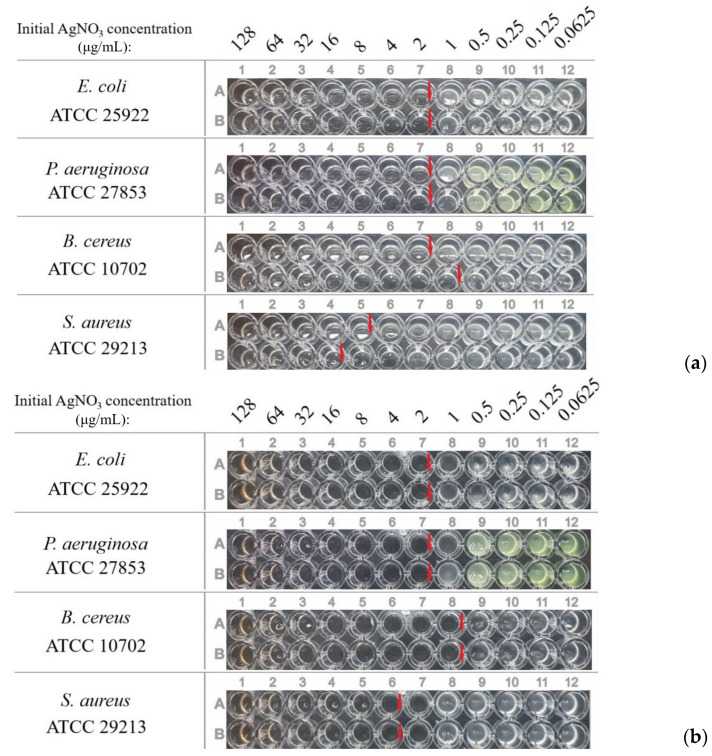
Plates with dispersions (α ≈ 45%) based on OChT-12/24-R (**a**) и OChT-12-25-R* (**b**).

The experiments showed that the minimum inhibitory concentration (MICs) values of these dispersions (Table 2, Figure 8) differed insignificantly and were within the margin of error of the method.

Comparing the obtained results with literature data [93,94,95,96,97,98,99], one can observe that the MICs of the synthesized silver NPs were higher than or comparable to those of other systems with silver NPs in most cases. One can see that *Staphylococcus aureus* ATCC 29213 was the least sensitive to dispersions because the concentrations required to inhibit its growth were the greatest.

**Figure 9:**
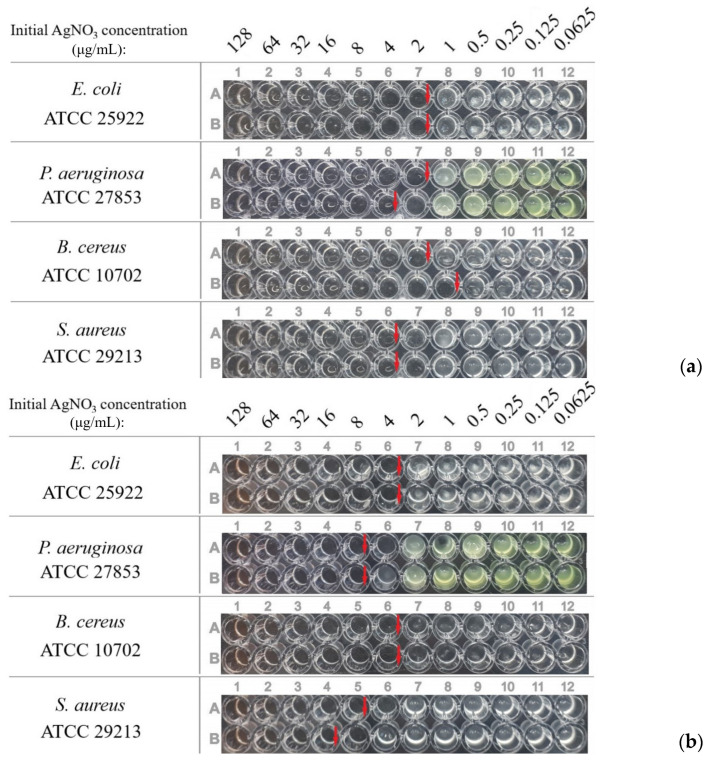
Plates with dispersions (α ≈ 100%) based on OChT-12/24-R (**a**) и OChT-12-25-R* (**b**).

To evaluate the influence of the presence of the silver ions, the MICs of dispersions with a reduction degree close to 100% were also determined (Table 3, Figure 9). There was no difference in the case of OChT-12/24-R. A low difference was for dispersions based on OChT-12/25-R*, but it was within the error of the method.

Thus, by the serial dilutions method, it was shown that silver nanodispersions based on OChT-12/24-R had antibacterial activity. The OChT itself did not contribute to the overall antibacterial activity of the dispersions. Dispersion MICs based on OChT-12/24-R and OChT-12/25-R* had no significant difference. Therefore, the presence of the silver chloride phase did not influence the effect nature of the total dispersion on the test microorganisms. Moreover, dispersion MICs with *α* ≈ 45% and close to 100% had no significant difference. So, it could be presumed that the activity of AgNP contributed the most to the overall effect of the dispersion rather than the precursor ions.

## 4. Conclusions

In this work, the kinetics of AgNP formation in an aqueous solution of N-reacetylated OChT hydrochloride was studied in detail. The complete reduction of silver ions occurred within 5 h and followed a typical pattern of kinetics of NP formation during chemical reduction with periods of induction, acceleration, and decay. The process is adequately described using the coordinates of the pseudo-first-order equation and is characterized by an effective constant of 0.012 min^−1^.

Chitosan acts as both a reducing agent and a stabilizer, so AgNPs are formed without the additional reducers and stabilizers. As a result of the process in an aqueous OChT hydrochloride solution, according to electron microscopy and DLS, individual particles of 10–25 nm are formed, which subsequently aggregate into larger aggregates of 60–90 nm in size, covered with a 2–4 nm stabilizing shell of chitosan. As a result, time-stable dispersions of NPs with a narrow monomodal distribution are formed. A silver chloride AgCl phase was detected by PXRD along with nanocrystalline silver in the obtained dispersions. It appears that the inorganic salt phase is formed in the early stages of AgNP synthesis.

It was demonstrated that the nanodispersions exhibited activity against both Gram-positive and Gram-negative bacteria. The dispersion MICs were 1–16 μg/mL, which is at least comparable to the literature data. By comparing the OChT-12/24-R based dispersion with the chloride-free OChT-12/25-R*, it was shown that the presence of AgCl did not significantly affect the dispersion activity. Moreover, no significant difference in the dispersion activity with half or full conversion was observed. Therefore, it can be inferred the AgNPs contributed the most to the dispersion activity rather than their precursor ions.

The nanodispersions, synthesized by a simple and reproducible method, exhibit antibacterial activity, and the AgNP chitosan stabilizing shell contains amine groups, which greatly facilitates their further functionalization. In that way, the systems are of interest for the design of materials focused on biomedical applications.

## Figures and Tables

**Table 1:** MICs of OChT-12/24-R and OChT-12/25-R* solutions.

	Culture			
	*Escherichia coli* ATCC 25922	*Pseudomonas aeruginosa* ATCC 27853	*Bacillus cereus* ATCC 10702	*Staphylococcus aureus* ATCC 29213
MICs of OChT-12/24-R solution, μg/mL	>48	>48	>48	>48
MICs of OChT-12/25-R* solution, μg/mL	>48	>48	>48	>48

MICs are the lowest concentration of agents that prevent any visible growth. The results of the experiments were reproducible. In cases of complete coincidence of the obtained data, MIC is represented as a single number.

**Table 2:** MICs of dispersions (α ≈ 45%) based on OChT-12/24-R and OChT-12/25-R*.

Culture	MICs of Dispersions (α ≈ 45%), μg/mL	
	Based on OChT-12/24-R	Based on OChT-12/25-R*
*Escherichia coli* ATCC 25922	2	2
*Pseudomonas aeruginosa* ATCC 27853	2	2
*Bacillus cereus* ATCC 10702	1–2	1
*Staphylococcus aureus* ATCC 29213	8–16	4

MICs are the lowest concentration of agents that prevent any visible growth. The results of the experiments were reproducible. In cases of complete coincidence of the obtained data, MIC is represented as a single number.

**Table 3:** MICs of dispersions (α ≈ 100%) based on OChT-12/24-R and OChT-12/25-R*.

Culture	MICs of Dispersions (α ≈ 100%), μg/mL	
	Based on OChT-12/24-R	Based on OChT-12/25-R*
*Escherichia coli* ATCC 25922	2	4
*Pseudomonas aeruginosa* ATCC 27853	2–4	8
*Bacillus cereus* ATCC 10702	1–2	4
*Staphylococcus aureus* ATCC 29213	4	8–16

MICs are the lowest concentration of agents that prevent any visible growth. The results of the experiments were reproducible. In cases of complete coincidence of the obtained data, MIC is represented as a single number.

## Data Availability

Data are contained within the article and Supplementary Materials.

## Supplementary Materials

The following supporting information can be downloaded at: https://www.mdpi.com/article/10.3390/pharmaceutics15122690/s1, Figure S1. Dependence of localized SPR peak maximum on inverse time.; Figure S2. SEM images of silver nanoparticles obtained by drying a drop of washed dispersion. Figure S3. EDX-spectra of initial OChT-12/24-R (a), initial OChT-12/25-R* (b) and AgNPs dispersion based on OChT-12/24-R (c). Figure S4. UV-visible spectrum of AgNPs dispersion based on OChT-12/25-R*. Figure S5. Particle size distributions of AgNPs dispersion based on OChT-12/25-R*. Figure S6. HRTEM images of AgNPs dispersion stabilized by OChT-12/25-R*. Table S1. PXRD qualitative analysis of samples.
