# Supplementary material for: Formation Kinetics and Antimicrobial Activity of Silver Nanoparticle Dispersions Based on N-Reacetylated Oligochitosan Solutions for Biomedical Applications
Source: Pharmaceutics. 2023 Nov 28;15(12):2690. doi: 10.3390/pharmaceutics15122690 (PMC10747331; doi:10.3390/pharmaceutics15122690)
Supplement: Supplementary file 1 [file pharmaceutics-15-02690-s001.zip › pharmaceutics-2684576-supplementary.pdf]

## Supplementary material

# Formation Kinetics and Antimicrobial Activity of Silver Nanoparticle Dispersions Based on N-Reacetylated Oligochitosan Solutions for Biomedical Applications

**Ekaterina K. Urodkova [1,*], Ol'ga Ya. Uryupina [1], Vladimir E. Tikhonov [2], Natalia E. Grammatikova [3], Anastasia V. Bol'shakova [1], Anna A. Sinelshchikova [1], Alexandra I. Zvyagina [1], Dmitry N. Khmelenin [4], Elena S. Zhavoronok [5] and Ivan N. Senchikhin [1,*]**

[1] A.N. Frumkin Institute of Physical Chemistry and Electrochemistry, Russian Academy of Sciences, 119071 Moscow, Russia; urupina635@mail.ru (O.Y.U.)
[2] A.N. Nesmeyanov Institute of Organoelement Compounds, Russian Academy of Sciences, 119991 Moscow, Russia; tikhon@ineos.ac.ru
[3] G.F. Gause Institute of New Antibiotics, 119021 Moscow, Russia; ngrammatikova@yandex.ru
[4] A.V. Shubnikov Institute of Crystallography, Russian Academy of Sciences, 119333 Moscow, Russia
[5] Lomonosov Institute of Fine Chemical Technologies, MIREA—Russian Technological University, 119571 Moscow, Russia
* Correspondence: urodkovakatja@mail.ru (E.K.U.); isenchikhin@gmail.com (I.N.S.)

Contents

Figure S1. Dependence of localized SPR peak maximum on inverse time.

Figure S2. SEM images of silver nanoparticles obtained by drying a drop of washed dispersion.

Table S1. PXRD qualitative analysis of samples.

Figure S3. EDX-spectra of initial OChT-12/24-R (*a*), initial OChT-12/25-R* (*b*) and AgNPs dispersion based on OChT-12/24-R (*c*).

Figure S4. UV-visible spectrum of AgNPs dispersion based on OChT-12/25-R*.

Figure S5. Particle size distributions of AgNPs dispersion based on OChT-12/25-R*.

Figure S6. HRTEM images of AgNPs dispersion stabilized by OChT-12/25-R*.

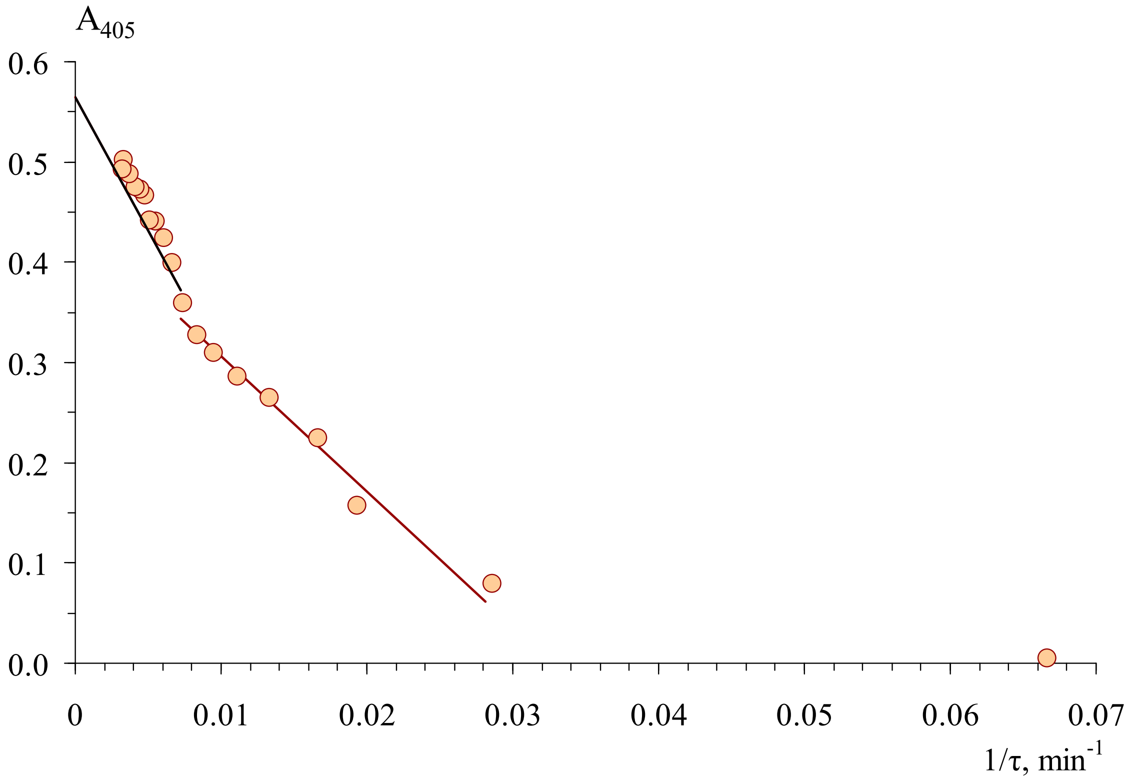


Figure S1. Dependence of LSPR peak maximum on inverse time.

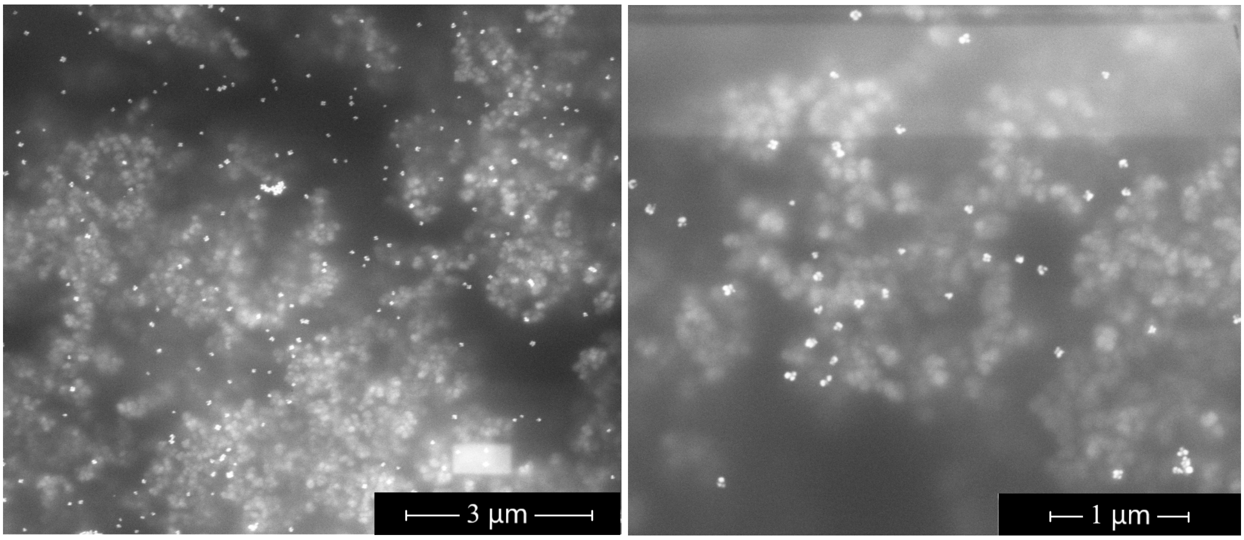


Figure S2. SEM images of silver nanoparticles obtained by drying a drop of washed dispersion on a silicon substrate.

Table S1. PXRD qualitative analysis of samples.

| **Experiment** | | | **The most close phase (PDF2)** | | | |
|---|---|---|---|---|---|---|
| ***d***, Å | Presence of a peak in the experimental diffractogram / ***I***, % | | **Space group** | **Cell parameters** | ***d***, Å / ***I**, %* | ***hkl*** |
| | **OChT-12/24-R** | **OChT-12/25-R*** | | | | |
| Phase I (Fig. 6) | | | Fm3m | a = 4.085 Å | Ag (4-783) | |
| 2.363 | + / 60 | + / 100 | | | 2.332 / 100 | 111 |
| 2.048 | + / 16 | + / 18 | | | 2.019 / 45 | 200 |
| 1.446 | + / 15 | + / 20 | | | 1.428 / 20 | 220 |
| 1.233 | + / 17 | + / 28 | | | 1.218 / 22 | 311 |
| Phase II (Fig. 6) | | | Fm3m | a = 5.549 Å | AgCl (31-1238) | |
| 3.210 | + / 46 | – | | | 3.200 / 50 | 111 |
| 2.780 | + / 100 | – | | | 2.770 / 100 | 200 |
| 1.965 | + / 52 | – | | | 1.962 / 60 | 220 |
| 1.675 | + / 13 | – | | | 1.670 / 18 | 311 |

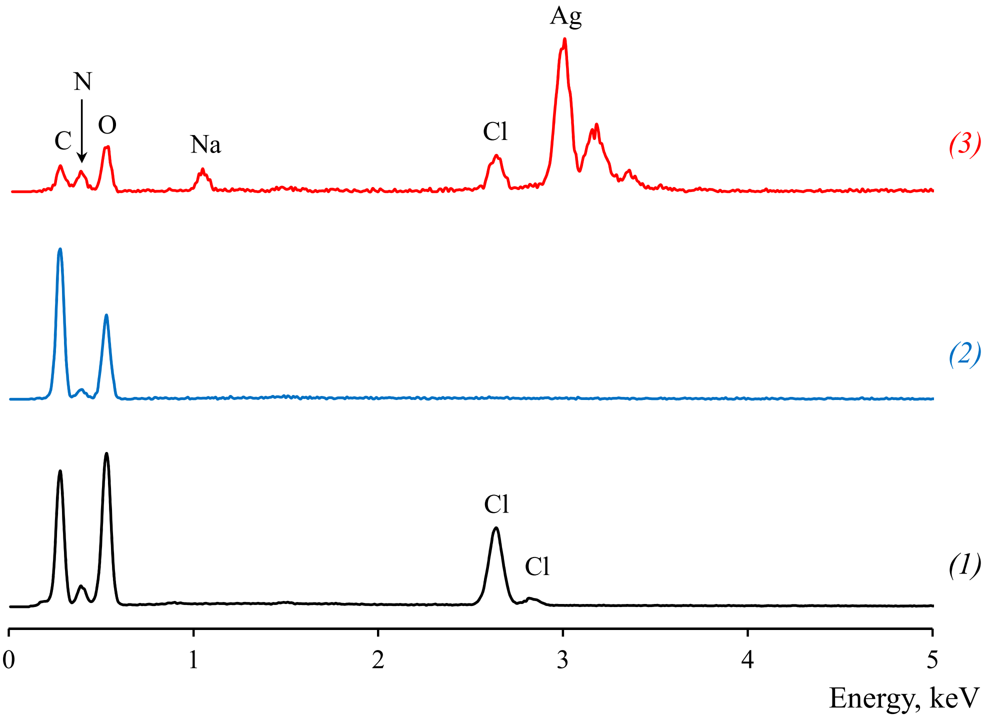


Figure S3. EDX-spectra of initial OChT-12/24-R (*1*), initial OChT-12/25-R* (*2*) and AgNPs dispersion based on OChT-12/24-R (*3*).

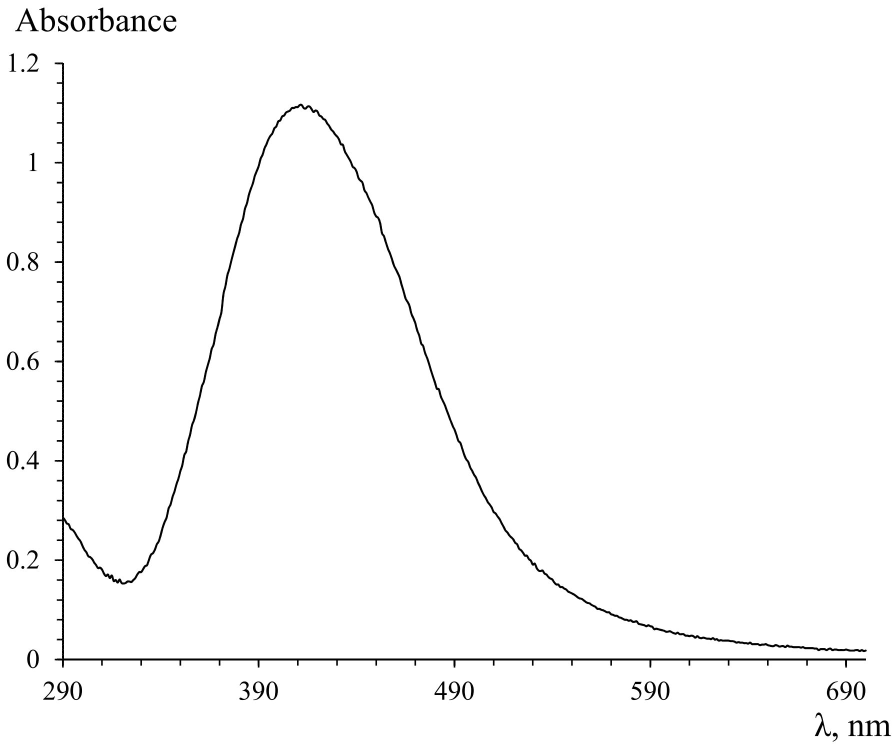


Figure S4. Typical UV-visible spectrum of dispersion based on OChT-12/25-R*.

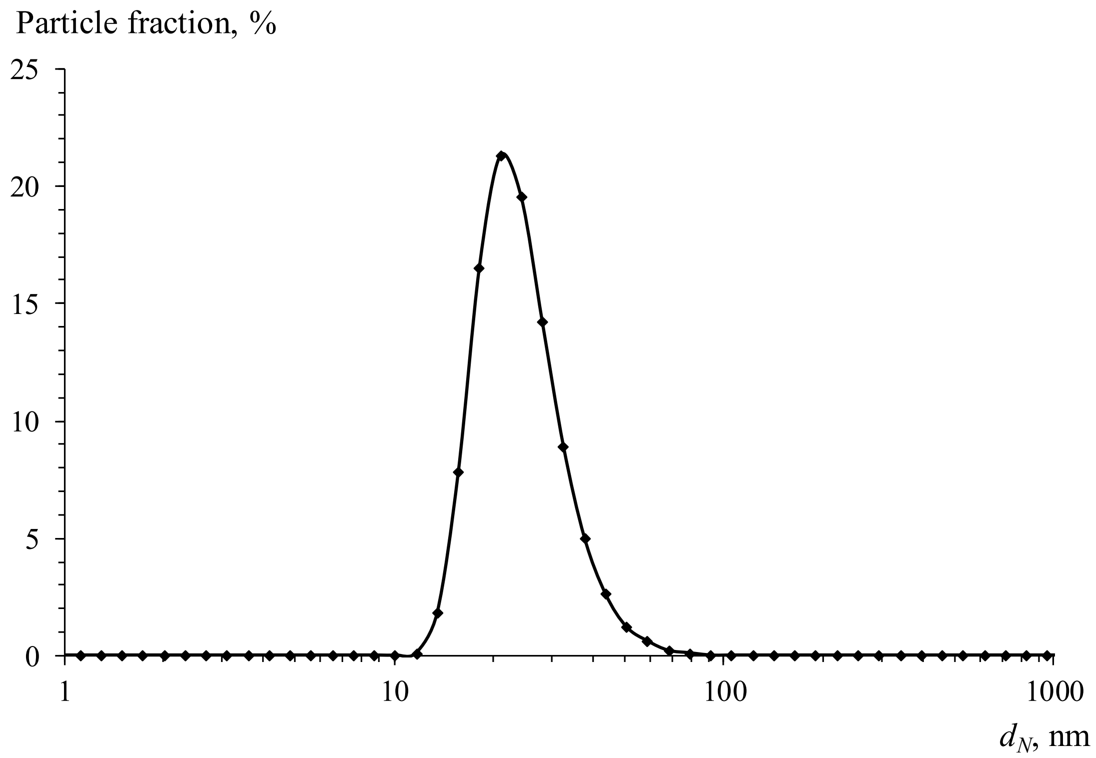


Figure S5. Typical particle size distributions of dispersion based on OChT-12/25-R*.

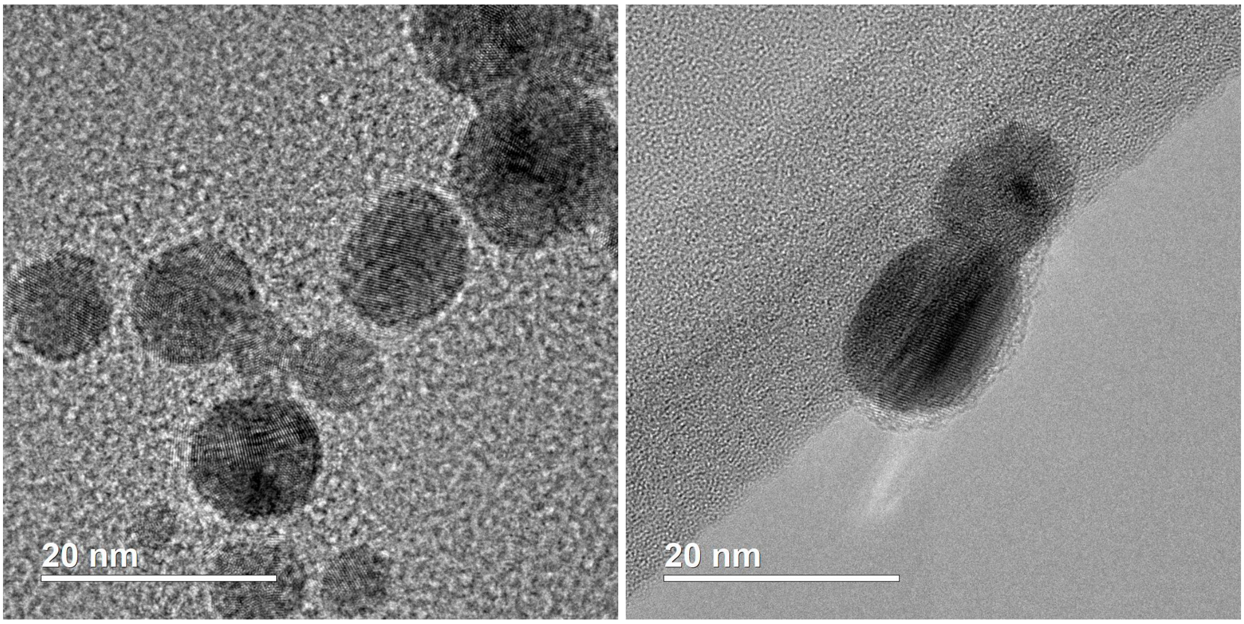


Figure S6. HRTEM images of dispersion stabilized by OChT-12/25-R*.
